# Silencing of long non-coding RNA NEAT1 improves Treg/Th17 imbalance in preeclampsia via the miR-485-5p/AIM2 axis

**DOI:** 10.1080/21655979.2021.1982306

**Published:** 2021-10-26

**Authors:** Jiying Chen, Yonggang Zhang, Wenqing Tan, Hanchao Gao, Shuixiu Xiao, Jinhua Gao, Zhiying Zhu

**Affiliations:** a Department of Obstetrics and Gynecology, Shenzhen Longhua District Central Hospital, Guangdong Medical University Affiliated Longhua District Central Hospital, Shenzhen, China; b Department of Clinical Laboratory, Shenzhen Longhua District Central Hospital, Guangdong Medical University Affiliated Longhua District Central Hospital, Shenzhen, China; c Department of General Practice, Shenzhen Longhua District Central Hospital, Guangdong Medical University Affiliated Longhua District Central Hospital, Shenzhen, China; d Department of Medical Laboratory, Shenzhen Longhua District Central Hospital, Guangdong Medical University Affiliated Longhua District Central Hospital, Shenzhen, China

**Keywords:** Preeclampsia, neat1, lncRNA, treg/th17, miR-485-5p/aim2 axis

## Abstract

T-regulatory (Treg)/T-helper 17 (Th17) imbalance is associated with preeclampsia (PE). Herein, we aimed to explore the effect and mechanism of lncRNA NEAT1 on the Treg/Th17 balance. The levels of nuclear enriched abundant transcript 1 (NEAT1), miR-485-5p, and absent in melanoma 2 (AIM2) in CD4^+^ T cells were determined using real-time quantitative polymerase chain reaction (RT-qPCR). Treg and Th17 cells were examined using flow cytometry. The relationship between miR-485-5p and NEAT1 or AIM2 was assessed using a dual-luciferase reporter assay. Pearson’s correlation coefficient was used to analyze the correlation. All the data indicated that NEAT1 was upregulated in PE. The number of Treg cells decreased and was negatively related to NEAT1, whereas the number of Th17 cells increased and was positively related to NEAT1 in PE. Knockdown of NEAT1 increased the Treg cells and Treg/Th17 but decreased Th17 cells. Furthermore, NEAT1 sponges miR-485-5p to suppress the target AIM2 levels. Inhibition of miR-485-5p or upregulation of AIM2 abrogated the effect on Treg/Th17 balance induced by knockdown of NEAT1. In conclusion, silencing of NEAT1 promoted Treg/Th17 balance via the miR-485-5p/AIM2 axis in PE, suggesting that NEAT1 is a potential target for the treatment of PE.

## Introduction

Preeclampsia (PE) is a common complication of pregnancy that manifests as multiple organ dysfunction [1]. It is defined as hypertension after 20 weeks of gestation with proteinuria of no less than 0.3 g/day [2]. PE can be divided into early or late onset, diagnosed before or after 34 weeks of gestation, respectively [3]. Globally, PE affects about 4% of pregnant women every year and may lead to perinatal maternal and fetal death [4]. Without timely, effective prevention and treatment, PE develops into more severe eclampsia with seizures [5]. However, because the pathogenesis of PE remains unclear, there is currently no complete cure for PE. The only clinical solution is to end the pregnancy and deliver the fetus and placenta [1]. Therefore, it is necessary to further explore the pathogenesis of PE and identify effective treatment strategies.

Long non-coding RNAs (lncRNAs) are transcripts with lengths of > 200 nt. They can only modulate gene expression in the form of RNA, and do not encode proteins. Recent studies have shown the involvement of lncRNAs in epigenetic regulation, chromatin modification, and transcriptional activation or interference [6]. Dysregulation of lncRNAs is implicated in the incidence and progression of diseases, such as cardiovascular diseases, degenerative diseases, diabetes, and cancer [7,8]. LncRNAs play significant roles in PE by regulating cellular processes, such as proliferation, differentiation, metastasis, and apoptosis [9,10]. The lncRNA NEAT1 is a structural part of paraspeckles and is crucial for RNA stability, isoform conversion, and paraspeckle assembly [11]. NEAT1 participates in the pathogenesis of malignancies [12]. However, the specific function of NEAT1 in PE has not yet been elucidated.

T-helper 17 (Th17) and T-regulatory (Treg) cells differentiate from T cells. Th17 cells promote inflammation through the production of IL-17, whereas Treg cells are specific suppressors of inflammation [13]. Balance between Tregs and Th17 cells is involved in immune homeostasis [14]. The differentiation of Treg and Th17 cells depends on the upregulation of Foxp3 in Tregs and RORγt in Th17 cells [15]. Treg/Th17 imbalance is associated with PE, which may be due to improper activation of the immune system during PE onset [16]. However, the mechanism of Treg/Th17 in PE remains unclear.

In this study, we aimed to explore the relationship between NEAT1 and Treg/Th17 balance in PE. We assumed that silencing of NEAT1 prevents Treg/Th17 imbalance in PE and the miR-485-5p/AIM2 axis is the molecular mechanism. These findings suggest that NEAT1 is a potential target for the treatment of PE.

## Materials and methods

### Subjects

This study was approved by the Shenzhen Longhua District Central Hospital (approval code No. 2,018,102,503). Written informed consent was obtained from each subject prior to the study. Participants included pregnant women (n = 25) diagnosed with PE, as well as 25 healthy pregnant women as a control. Subjects enrolled in our study were natural singleton pregnancy. Patients with the following diseases were excluded from this study: autoimmune diseases, pre-pregnancy chronic hypertension, cancer, and other pregnancy complications. Whole blood samples were collected from all subjects.

### Cell collection and culture

Peripheral blood mononuclear cells (PBMCs) were isolated from the blood of healthy pregnant women and patients with PE. The isolation was performed using Ficoll–Hypaque with density gradient centrifugation. Venous blood (2 mL) collected from all subjects was mixed with Hank’s solution (2 mL). After adding 2 mL Ficoll–Hypaque solution (MP Biomedicals, Aurora, OH, USA), the samples were centrifuged at 2000 r/min for 5 min. Next, the mononuclear cell layer was transferred to another centrifuge tube, mixed with Hank’s solution, and centrifuged at 2000 r/min for 5 min. After washing with Hank’s solution, PBMCs were maintained in PBMC complete medium (Procell, Wuhan, China) at 37°C with 5% CO_2_.

CD4^+^ T cells were isolated from PBMCs using the EasySep™ Human CD4^+^ T Cell Isolation Kit (StemCell Technologies, Vancouver, BC, Canada). Briefly, 5 х 10^7^ cells were incubated with cocktail (50 μL) for 5 min and the samples were mixed with RapidSpheres (50 μL) and the EasySep™ Buffer was added up to 2.5 mL. The samples were then incubated with a magnet for 3 min at 25°C. After removing the magnet, an enriched cell suspension remained.

### Bioinformatic analysis

Microarray analysis was performed on the Human LncRNA + mRNA Array 4.0 platform using R language. LncRNAs (Log_2_ ≥ 2 and P < 0.05) were chosen to create a heatmap. The targets of NEAT1 or miR-485-5p were predicted using the Starbase (http://starbase.sysu.edu.cn/starbase2/index.php) or TargetScan (http://www.targetscan.org/vert_72/) online tool.

### Reverse transcription-quantitative PCR (RT-qPCR)

Total RNA was isolated using TRNzol Universal Reagent (TIANGEN, Beijing, China). To detect NEAT1 and AIM2, RT and qPCR were conducted using the FastKing One Step RT-qPCR Kit (SYBR Green) (TIANGEN) following the manufacturer’s protocol. The reaction conditions of RT-qPCR were 50°C for 30 min, 95°C for 3 min, 40 cycles of 95°C for 15 sec and 60°C for 30 sec. The reaction system (50 μL) of RT-qPCR includes 1х FastKing RT-qPCR Buffer (25 μL), 1× RT-PCR Enzyme Mix (2 μL), 10 μM forward primer (1.25 μL), 10 μM reverse primer (1.25 μL), 1 μg total RNA (10 μL), and Rnase-free ddH_2_O (10.5 μL). *GAPDH* was used for normalization. To examine miR-485-5p, RT was conducted using the miRcute miRNA First-Strand cDNA Synthesis Kit (TIANGEN) at 37°C for 60 min after treating with Poly(A). qPCR was performed using the miRcute miRNA qPCR Detection Kit (TIANGEN) with the following conditions: 94°C for 2 min, 40 cycles of 94°C for 20 sec and 60°C for 34 sec. The reaction system (20 μL) of qPCR includes 1х miRcute miRNA Premix (10 μL), 200 nM forward primer (0.4 μL), 200 nM reverse primer (0.4 μL), First-Strand cDNA (2 μL), and RNsae-free ddH_2_O (7.2 μL). *U6* was used for normalization. qPCR was performed using a Line-Gene Real Time PCR system (Bioer, Hangzhou, China). Relative expression was analyzed using the 2^−ΔΔCT^ method.

### Cell transfection

Small interfering RNA negative control (si-NC), si-NEAT1#1, si-NEAT1#2, miR-485-5p inhibitor, inhibitor NC, pcDNA3.1, and pcDNA3.1-AIM2 were acquired from GenePharma (Shanghai, China). Cell transfection in CD4^+^ T cells isolated from patients with PE was assessed using the Human T Cell Nucleofector Kit on an Amaxa 4D-Nucleofector Electroporation system (Lonza, Basel, Switzerland) for 48 h.

### Flow cytometry

Cells were stained with FITC-conjugated anti-mouse CD4 and then permeabilized with Foxp3 staining buffer. Next, the cells were stained with PE-conjugated anti-mouse Foxp3 (0.125 μg) or PE-conjugated anti-mouse RORγt (0.125 μg) and kept in the dark at 4°C for 40 min. Antibodies were obtained from eBioscience (San Diego, CA, USA). Flow cytometry was performed using a CytoFLEX (V2-B2-R2) instrument (Beckman Coulter, Fullerton, CA, USA) [17].

### Dual-luciferase reporter assay

Wild type (WT) and mutant sequences of NEAT1 and AIM2 were cloned into the Pmir-Glo vector (Promega, Madison, USA) to construct the recombinant plasmids. HEK293T cells were co-transfected with mimics or NC and WT or mutant (MUT) reporter plasmids using Lipofectamine 2000 (Invitrogen, Carlsbad, USA) for 48 h. The fluorescence signals were measured using the Luciferase Assay System (Promega).

### Western blot

Total protein was isolated using RIPA lysis buffer (Beyotime, Shanghai, China). After testing the protein concentration using BCA Kit (TIANGEN), each protein sample was separated using 10% SDS-PAGE and transferred onto PVDF membranes. After blocking using 5% nonfat milk, the membranes were incubated with anti-AIM2 at 4°C overnight and then incubated with secondary antibody at 25°C for 2 h. Protein bands were visualized using ECL reagent (Beyotime) and quantified using Image J software (version 1.8.0; National Institutes of Health, Bethesda, Maryland, USA).

### Statistical analysis

Data were analyzed using GraphPad Prism 8.0 software (San Diego, USA) and are shown as mean ± SD. Student’s *t*-test and one-way ANOVA were used to evaluate the significance between and among groups, respectively. Pearson’s correlation coefficient was used to analyze the correlation.

## Results

The present study aimed to investigate the the effect of NEAT1 on Treg/Th17 balance in PE and further revealed the potential molecular mechanism. In this study, we found a loss of NEAT1 improves Treg/Th17 imbalance by regulating the miR-485-5p/AIM2 axis in PE. This study may provide a new insight to treat PE.

### NEAT1 is upregulated in PE and associated with Treg/Th17 balance

**Figure 1.**
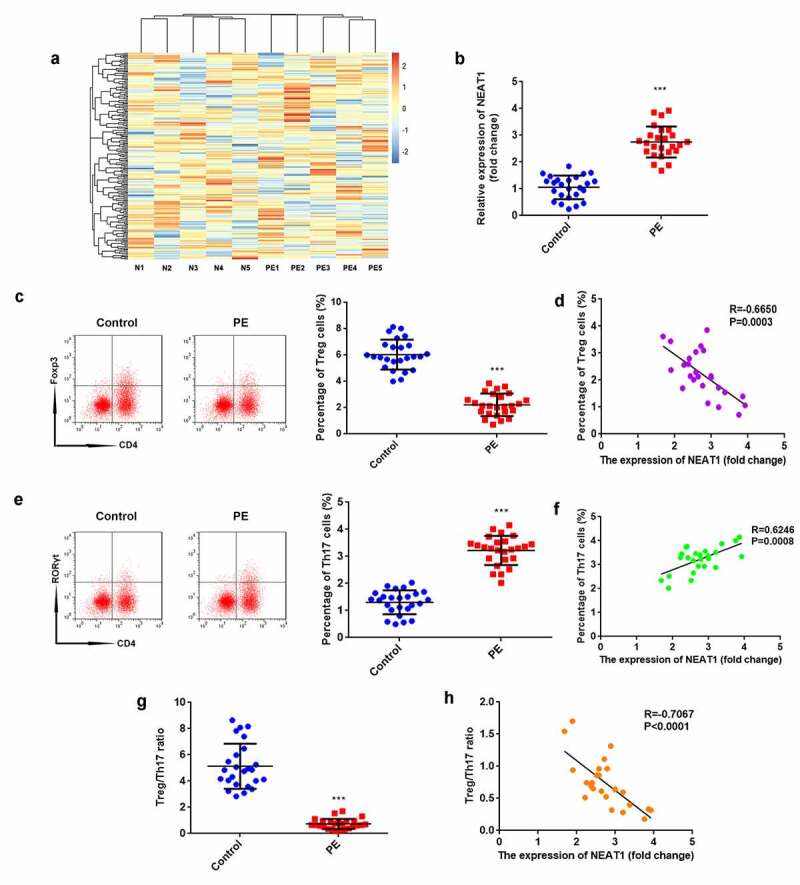
NEAT1 expression is upregulated in PE and associated with Treg/Th17 balance. (a) heatmap analysis showing lncRNA expression profile in patients with PE and healthy pregnant women. (b) the levels of NEAT1 were examined using RT-qPCR in CD4^+^ T cells isolated from patients with PE and healthy pregnant women. (c) Treg cells were assessed by flow cytometry and quantified. (d) correlation analysis evaluated the interaction between NEAT1 and Treg cells. (e) Th17 cells were analyzed using flow cytometry and quantified. (f) Correlation analysis between NEAT1 and Th17 cells. (g) calculation of the ratio of Treg/Th17. (h) correlation analysis evaluated the relationship between NEAT1 and Treg/Th17 ratio. ***P < 0.001

According to the microarray results, NEAT1 expression was predicted to be higher in patients with PE than in healthy pregnant women (Figure 1(a)). The results of RT-qPCR proved that NEAT1 expression was significantly elevated in the PE group (P < 0.001; Figure 1(b)). The percentage of Treg cells significantly decreased in PE (P < 0.001), which negatively correlated with NEAT1 levels (P = 0.0003, R = −0.6650; Figure 1(c-d)). In contrast, the percentage of Th17 cells significantly increased in PE (P < 0.001), which positively correlated with NEAT1 levels (P = 0.0008, R = 0.6246; Figure 1(e-f)). The ratio of Treg/Th17 was significantly reduced in patients with PE (P < 0.001), which negatively correlated to NEAT1 levels (P < 0.0001, R = −0.7067; Figure 1(g-h)).

### Knockdown of NEAT1 promotes the balance of Treg/Th17

**Figure 2.**
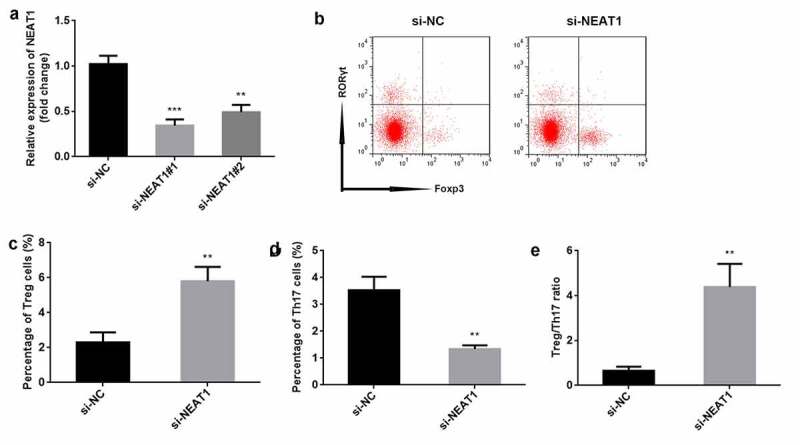
Knockdown of NEAT1 promotes the balance of Treg/Th17. (a) transfection efficiency was determined using RT-qPCR. (b) Treg and Th17 cells were assessed by flow cytometry. the percentage of (c) Treg cells and (d) Th17 cells was quantified. (e) the ratio of Treg/Th17 was calculated. ***P < 0.001. **P < 0.01

To explore the role of NEAT1, the levels of NEAT1 were markedly decreased after transfection with si-NEAT#1 (P < 0.001) and si-NEAT#2 (P < 0.01), particularly si-NEAT#1 (Figure 2(a)). si-NEAT#1 was therefore used in this study. Knockdown of *NEAT1* resulted in increased number of Treg cells (P < 0.01), and decreased number of Th17 cells (P < 0.01) and Treg/Th17 ratio (P < 0.01), compared to the si-NC group (Figure 2(b–e)).

### NEAT1 acts as a miR-485-5p sponge

**Figure 3.**
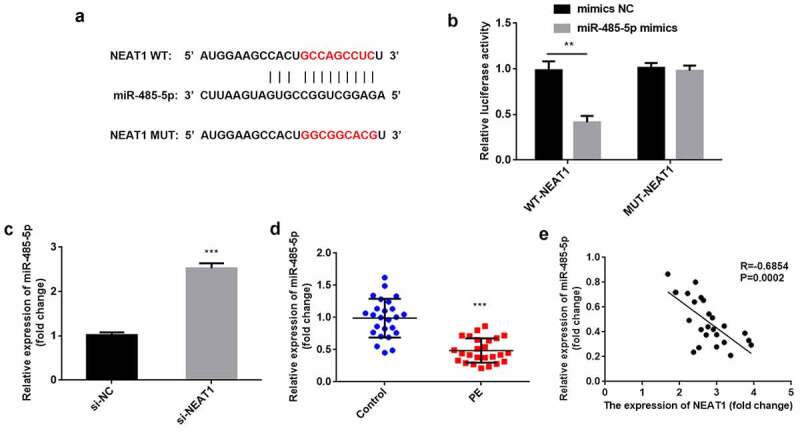
NEAT1 acts as a miR-485-5p sponge. the interaction between NEAT1 and miR-485-5p was (a) predicted using the starbase dataset and (b) confirmed using dual-luciferase reporter analysis. (c) miR-485-5p levels were detected by RT-qPCR with knockdown of NEAT1. (d) miR-485-5p was measured in CD4^+^ T cells isolated from patients with PE and healthy pregnant women. (e) Correlation analysis between NEAT1 and miR-485-5p in PE. ***P < 0.001. **P < 0.01

Next, the NEAT1 targets were predicted. We found the binding sites of the NEAT1 3′-UTR in miR-485-5p (Figure 3(a)). miR-485-5p mimics significantly reduced relative luciferase activity in the WT-NEAT1 group but not in the MUT-NEAT1 group, compared with the NC group (P < 0.01; Figure 3(b)). After knockdown of NEAT1, miR-485-5p expression was significantly increased ((P < 0.001; Figure 3(c)). In PE, miR-485-5p was downregulated (P < 0.001), which negatively correlated with NEAT1 levels (P = 0.0002, R = −0.6854; Figure 3(d-e)).

### Knockdown of NEAT1 improves Treg/Th17 imbalance via sponging miR-485-5p

**Figure 4.**
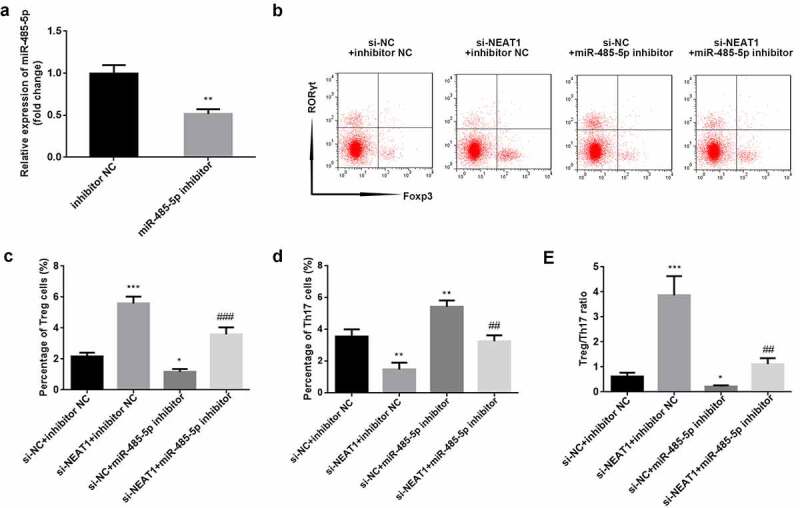
Knockdown of NEAT1 improves Treg/Th17 imbalance via sponging of miR-485-5p. (a) transfection efficiency was examined by RT-qPCR in CD4^+^ T cells following transfection with inhibitor NC or miR-485-5p inhibitor. (b) Treg and Th17 cells were assessed using flow cytometry. (c) quantification of the percentage of Treg cells. (d) quantification of the percentage of Treg cells. (e) calculation of the ratio of Treg/Th17. **P < 0.01 vs. inhibitor NC group in (A). ***P < 0.001, **P < 0.01 and *P < 0.05 vs. si-NC + inhibitor NC group. ###P < 0.01 and ##P < 0.01 vs. si-NEAT1 + inhibitor NC group

As illustrated in (Figure 4(a)), miR-485-5p was significantly downregulated when CD4^+^ T cells were transfected with the miR-485-5p inhibitor (P < 0.01; Figure 4(a)). Functionally, inhibition of miR-485-5p reversed the increase in Treg and the decrease in Th17 cells induced by knockdown of NEAT1 (Treg: P < 0.001; Th17: P < 0.01; Figure 4(b–d)). Moreover, knockdown of NEAT1 enhanced (P < 0.001), but treatment with miR-485-5p inhibitor markedly reduced the Treg/Th17 ratio (P < 0.05). The miR-485-5p inhibitor abolished the increase in Treg/Th17 ratio induced by si-NEAT1 (P < 0.01; Figure 4(e)).

### AIM2 is a miR-485-5p downstream target

**Figure 5.**
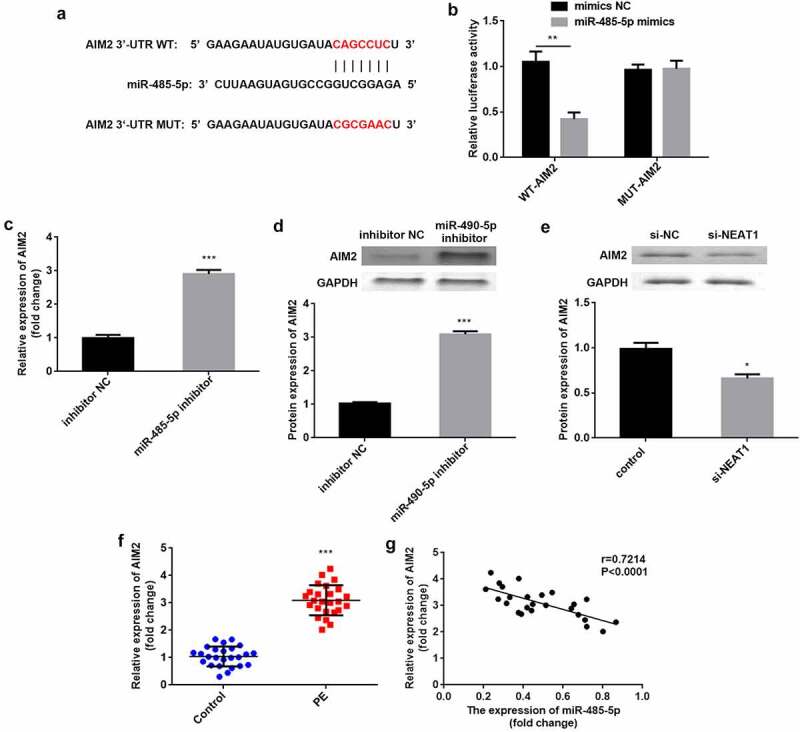
AIM2 is a miR-485-5p target. (a) the predicted binding sites of AIM2 in miR-485-5p were analyzed using the targetscan dataset. (d) the luciferase activity was assessed when co-transfected with WT-AIM2 or MUT-AIM2 and mimics or NC. (c) AIM2 was tested by RT-qPCR when inhibiting miR-485-5p. (D) AIM2 protein expression was detected using western blot when inhibiting miR-485-5p. (e) AIM2 protein levels were measured by western blot when knockdown of NEAT1. (f) AIM2 was measured in CD4^+^ T cells isolated from patients with PE and healthy pregnant women. (g) Correlation analysis between AIM2 and miR-485-5p expression in PE. ***P < 0.001. **P < 0.01

As illustrated in (Figure 5(a)), AIM2 in the 3′-UTR region has binding sites for miR-485-5p. Compared with NC, luciferase activity was significantly suppressed when co-transfected with mimics and WT-AIM2, but was not influenced by co-transfection with mimics and MUT-AIM2 (P < 0.01; Figure 5(b)). The mRNA and protein levels of AIM2 were significantly elevated by the inhibition of miR-485-5p (P < 0.001; Figure 5(c-d)). The protein levels of AIM2 were significantly reduced by knockdown of NEAT1 (P < 0.05; Figure 5(e)). Additionally, AIM2 levels were higher in patients with PE than in healthy subjects (P < 0.001), and negatively correlated with miR-485-5p (P < 0.0001, R = 0.7214; figure 5(f-g)).

### Knockdown of NEAT1 improves Treg/Th17 imbalance through modulation of AIM2 expression

**Figure 6.**
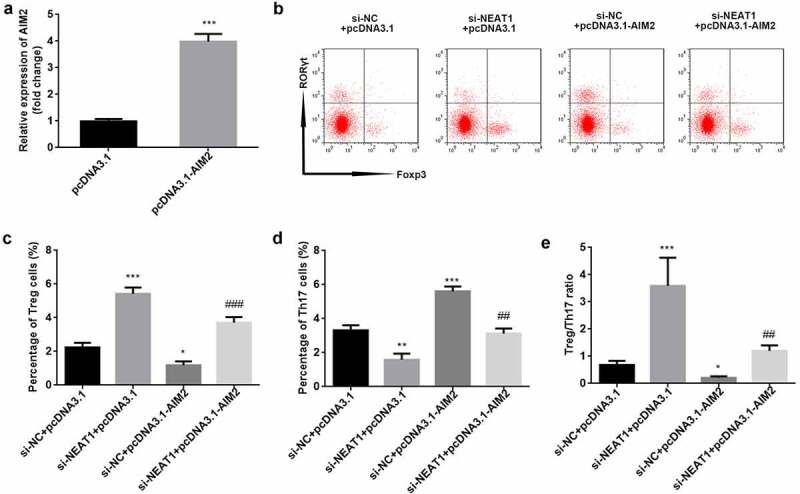
Knockdown of NEAT1 improves Treg/Th17 imbalance through modulation of AIM2. (a) Transfection efficiency was examined by RT-qPCR in CD4^+^ T cells following transfection of pcDNA3.1 or pcDNA3.1-AIM2. (b) Treg and Th17 cells were assessed using flow cytometry. the percentage of (c) Treg cells and (d) Th17 cells was quantified. (e) calculation of the ratio of Treg/Th17. ***P < 0.001 vs. pcDNA3.1 group in (A). ***P < 0.001, **P < 0.01 and *P < 0.05 vs. si-NC + pcDNA3.1 group. ###P < 0.01 and ##P < 0.01 vs. si-NEAT1 + pcDNA3.1 group

AIM2 levels were significantly higher in the pcDNA3.1-AIM2 group than in the pcDNA3.1 one (P < 0.001; Figure 6(a)). The results of flow cytometry showed that overexpression of AIM2 abolished the increased number of Treg cells and decreased number of Th17 cells induced by NEAT1 knockdown (Treg: P < 0.001; Th17: P < 0.01; Figure 6(b–d)). The Treg/Th17 balance was markedly promoted by knockdown of NEAT1, while overexpression of AIM2 reversed this effect (P < 0.01; Figure 6(e)).

## Discussion

In this study, we first observed that NEAT1 silencing promoted the balance of Treg/Th17 cells in PE. Furthermore, the miR-485-5p/AIM2 axis was first identified as the underlying mechanism.

Abnormal activation of the maternal immune system causes placental dysfunction, leading to the pathogenesis of PE, followed by a systemic inflammatory response [3,18]. Thus, Treg/Th17 imbalance is a mechanism of PE, manifesting as impaired Treg activity and excessive Th17 response [19]. Studies have reported that inducing an imbalance in Treg/Th17 using low vitamin D, CD81, and alteration of PD-1/PD-L1 contributes to PE [20–22]. In this study, we found that the percentage of Treg cells decreased, but that of Th17 increased, proving that Treg/Th17 imbalance is involved in PE.

NEAT1 is a newly identified immune regulator that affects mononuclear-macrophage function as well as T cell differentiation [23]. During the immune response, NEAT1 can promote the activation of inflammasomes, such as NLRP3 and NLRC4 in macrophages [24]. Additionally, NEAT1 inhibits the inflammatory response by regulating macrophage polarization [25]. A previous study revealed that knockdown of NEAT1 inhibits Th17/CD4^+^ T cell differentiation [26]. However, the effects of NEAT1 on Treg/Th17 balance remain unknown. NEAT1 is upregulated in the placenta of rats with PE and inhibits the growth of trophoblast cells [27]. Herein, we found that NEAT1 was increased in patients with PE, consistent with results found in the rat placenta. Moreover, we found that knockdown of NEAT1 improved Treg/Th17 imbalance, providing a new insight into NEAT1 function in PE.

LncRNAs exert sponge or decoy functions to reduce the effects of miRNAs on mRNAs [28]. To investigate the molecular mechanism, miR-485-5p was used as a NEAT1 target. Studies have indicated that miR-485-5p has a tumor-suppressive function [29,30]. Additionally, miR-485-5p is associated with inflammation or immune response. For example, miR-485-5p produces inflammatory factors and suppresses cell differentiation to promote the development of osteoarthritis [31]. Nevertheless, downregulation of miR-485-5p promotes inflammatory pain [32]. Additionally, miRNAs can regulate Th17 cell differentiation and thus influence Treg/Th17 balance, such as miR-146a-5p [33]. In our study, miR-485-5p was downregulated in PE and negatively related to NEAT1 expression. Moreover, inhibition of miR-485-5p abolished the improved Treg/Th17 balance induced by the knockdown of NEAT1.

AIM2, an innate immune sensor, can identify DNA from microbes and hosts, and is arguably a specific marker of innate immune initiation [34,35]. The activation of AIM2 causes the secretion of pro-inflammatory cytokines and induces cell pyroptosis [36]. The AIM inflammasome responds to various diseases, such as skin disease, diabetes, cancer, and infectious diseases [37]. However, the involvement of AIM2 in Treg/Th17 balance remains unknown. In the current study, AIM2 was found to be a target of miR-485-5p. The level of AIM2 was increased in PE, consistent with a previous study [38]. Furthermore, overexpression of AIM2 abolished the effects on Treg/Th17 balance induced by knockdown of *NEAT1*. Taken together, these findings suggest that silencing of NEAT1 improves Treg/Th17 imbalance by sponging miR-485-5p.

## Conclusion

NEAT1 expression was upregulated in PE and associated with a Treg/Th17 imbalance. Furthermore, silencing of NEAT1 promoted the balance of Treg/Th17 cells by modulating the miR-485-5p/AIM2 axis. These findings suggest that NEAT1 is a potential target for the treatment of PE.

## RESEARCH HIGHLIGHTS

1. LncRNA NEAT1 is associated with Treg/Th17 imbalance in PE.
2. NEAT1 is a sponge of miR-485-5p to upregulated AIM2 expression.
3. NEAT1 promoted Treg/Th17 balance via miR-485-5p/AIM2 axis in PE.

## References

[1] Bokslag A, van Weissenbruch M, Mol BW, et al. Preeclampsia; short and long-term consequences for mother and neonate. Early Hum Dev. 2016;102:47–50. 27659865 10.1016/j.earlhumdev.2016.09.007

[2] El-Sayed AAF. Preeclampsia: a review of the pathogenesis and possible management strategies based on its pathophysiological derangements. Taiwan J Obstet Gynecol. 2017;56(5):593–598. 29037542 10.1016/j.tjog.2017.08.004

[3] Aneman I, Pienaar D, Suvakov S, et al. Mechanisms of key innate immune cells in early- and late-onset preeclampsia. Front Immunol. 2020;11:1864. 33013837 10.3389/fimmu.2020.01864 PMC7462000

[4] Mol BWJ, Roberts CT, Thangaratinam S, et al. Pre-eclampsia. Lancet. 2016;387(10022):999–1011. 26342729 10.1016/S0140-6736(15)00070-7

[5] Jido TA, Yakasai IA. Preeclampsia: a review of the evidence. Ann Afr Med. 2013;12(2):75–85. 23713013 10.4103/1596-3519.112395

[6] Li X, Wu Z, Fu X, et al. lncRNAs: insights into their function and mechanics in underlying disorders. Mutat Res Rev Mutat Res. 2014;762:1–21. 25485593 10.1016/j.mrrev.2014.04.002

[7] Schmitz SU, Grote P, Herrmann BG. Mechanisms of long noncoding RNA function in development and disease. Cell Mol Life Sci. 2016;73(13):2491–2509. 27007508 10.1007/s00018-016-2174-5 PMC4894931

[8] Ransohoff JD, Wei Y, Khavari PA. The functions and unique features of long intergenic non-coding RNA. Nat Rev Mol Cell Biol. 2018;19(3):143–157. 29138516 10.1038/nrm.2017.104 PMC5889127

[9] Jin X, Ma X, Zhu Y. Investigating Dysregulated Sub-Pathways for Preeclampsia Infants Based on lncRNA-mRNA Expression Data and Pathway Network. Ann Clin Lab Sci. 2019;49(5):598–607. 31611202

[10] Yang X, Meng T. Long Noncoding RNA in Preeclampsia: transcriptional Noise or Innovative Indicators? Biomed Res Int. 2019;2019:5437621. 31111058 10.1155/2019/5437621 PMC6487157

[11] Yamazaki T, Souquere S, Chujo T, et al. Functional Domains of NEAT1 Architectural lncRNA Induce paraspeckle assembly through phase separation. Mol Cell. 2018;70(6):1038–1053.e7. 29932899 10.1016/j.molcel.2018.05.019

[12] Ghafouri-Fard S, Taheri M. Nuclear Enriched Abundant Transcript 1 (NEAT1): a long non-coding RNA with diverse functions in tumorigenesis. Biomed Pharmacother. 2019;111:51–59. 30576934 10.1016/j.biopha.2018.12.070

[13] Omenetti S, Pizarro TT. The Treg/Th17 Axis: a Dynamic Balance Regulated by the Gut Microbiome. Front Immunol. 2015;6:639. 26734006 10.3389/fimmu.2015.00639 PMC4681807

[14] Kimura A, Kishimoto T. IL-6: regulator of Treg/Th17 balance. Eur J Immunol. 2010;40(7):1830–1835. 20583029 10.1002/eji.201040391

[15] Zhou L, Lopes JE, Chong MM, et al. TGF-beta-induced Foxp3 inhibits T(H)17 cell differentiation by antagonizing RORgammat function. Nature. 2008;453(7192):236–240. 18368049 10.1038/nature06878 PMC2597437

[16] Chang GP, Yang XL, Liu W, et al. FABP4 facilitates inflammasome activation to induce the Treg/Th17 imbalance in preeclampsia via forming a positive feedback with IL-17A. Mol Ther Nucleic Acids. 2021;24:743–754. 33996256 10.1016/j.omtn.2021.03.020 PMC8094592

[17] Chi X, Guo Y, Zhang L, et al. Long non-coding RNA GAS5 regulates Th17/Treg imbalance in childhood pneumonia by targeting miR-217/STAT5. Cell Immunol. 2021;364:104357. 33862314 10.1016/j.cellimm.2021.104357

[18] Rambaldi MP, Weiner E, Mecacci F, et al. Immunomodulation and preeclampsia. Best Pract Res Clin Obstet Gynaecol. 2019;60:87–96. 31311760 10.1016/j.bpobgyn.2019.06.005

[19] Jafri S, Ormiston ML. Immune regulation of systemic hypertension, pulmonary arterial hypertension, and preeclampsia: shared disease mechanisms and translational opportunities. Am J Physiol Regul Integr Comp Physiol. 2017;313(6):R693–R705. 28978513 10.1152/ajpregu.00259.2017

[20] Muyayalo KP, Huang XB, Qian Z, et al. Low circulating levels of vitamin D may contribute to the occurrence of preeclampsia through deregulation of Treg/Th17 cell ratio. Am J Reprod Immunol. 2019;82(4):e13168. 31299118 10.1111/aji.13168

[21] Ding H, Dai Y, Lei Y, et al. Upregulation of CD81 in trophoblasts induces an imbalance of Treg/Th17 cells by promoting IL-6 expression in preeclampsia. Cell Mol Immunol. 2019;16(4):302–312. 30487550 10.1038/s41423-018-0186-9 PMC6318306

[22] Zhang Y, Liu Z, Tian M, et al. The altered PD-1/PD-L1 pathway delivers the ‘one-two punch’ effects to promote the Treg/Th17 imbalance in pre-eclampsia. Cell Mol Immunol. 2018;15(7):710–723. 28890543 10.1038/cmi.2017.70 PMC6123412

[23] Gast M, Rauch BH, Haghikia A, et al. Long noncoding RNA NEAT1 modulates immune cell functions and is suppressed in early onset myocardial infarction patients. Cardiovasc Res. 2019;115(13):1886–1906. 30924864 10.1093/cvr/cvz085

[24] Zhang P, Cao L, Zhou R, et al. The lncRNA Neat1 promotes activation of inflammasomes in macrophages. Nat Commun. 2019;10(1):1495. 30940803 10.1038/s41467-019-09482-6 PMC6445148

[25] Liu R, Tang A, Wang X, et al. Inhibition of lncRNA NEAT1 suppresses the inflammatory response in IBD by modulating the intestinal epithelial barrier and by exosome-mediated polarization of macrophages. Int J Mol Med. 2018;42(5):2903–2913. 30132508 10.3892/ijmm.2018.3829

[26] Shui X, Chen S, Lin J, et al. Knockdown of lncRNA NEAT1 inhibits Th17/CD4(+) T cell differentiation through reducing the STAT3 protein level. J Cell Physiol. 2019;234(12):22477–22484. 31119756 10.1002/jcp.28811

[27] Teng L, Liu P, Song X, et al. Long Non-Coding RNA Nuclear-Enriched Abundant Transcript 1 (NEAT1) Represses Proliferation of Trophoblast Cells in Rats with Preeclampsia via the MicroRNA-373/FLT1 Axis. Med Sci Monit. 2020;26:e927305. 33093438 10.12659/MSM.927305 PMC7590520

[28] Paraskevopoulou MD, Ag H. Analyzing MiRNA-LncRNA Interactions. Methods Mol Biol. 2016;1402:271–286. 26721498 10.1007/978-1-4939-3378-5_21

[29] Pan Y, Qin J, Sun H, et al. MiR-485-5p as a potential biomarker and tumor suppressor in human colorectal cancer. Biomark Med. 2020;14(3):239–248. 31984757 10.2217/bmm-2019-0534

[30] Wang FR, Xu SH, Wang BM, et al. MiR-485-5p inhibits metastasis and proliferation of osteosarcoma by targeting CX3CL1. Eur Rev Med Pharmacol Sci. 2018;22(21):7197–7204. 30468463 10.26355/eurrev_201811_16253

[31] Chen HO, Zhang L, Tang ZY, et al. MiR-485-5p promotes the development of osteoarthritis by inhibiting cartilage differentiation in BMSCs. Eur Rev Med Pharmacol Sci. 2018;22(11):3294–3302. 29917178 10.26355/eurrev_201806_15148

[32] Xu M, Wu R, Zhang L, et al. Decreased MiR-485-5p Contributes to Inflammatory Pain Through Post-Transcriptional Upregulation of ASIC1 in Rat Dorsal Root Ganglion. J Pain Res. 2020;13:3013–3022. 33239909 10.2147/JPR.S279902 PMC7682601

[33] Wang X, Xin S, Wang Y, et al. MicroRNA-146a-5p enhances T helper 17 cell differentiation via decreasing a disintegrin and metalloprotease 17 level in primary sjogren’s syndrome. Bioengineered. 2021;12(1):310–324. 33446013 10.1080/21655979.2020.1870321 PMC8806215

[34] Lugrin J, Martinon F. The AIM2 inflammasome: sensor of pathogens and cellular perturbations. Immunol Rev. 2018;281(1):99–114. 29247998 10.1111/imr.12618

[35] Man SM, Karki R, Kanneganti TD. AIM2 inflammasome in infection, cancer, and autoimmunity: role in DNA sensing, inflammation, and innate immunity. Eur J Immunol. 2016;46(2):269–280. 26626159 10.1002/eji.201545839 PMC4758349

[36] Wang B, Bhattacharya M, Roy S, et al. Immunobiology and structural biology of AIM2 inflammasome. Mol Aspects Med. 2020;76:100869. 32660715 10.1016/j.mam.2020.100869 PMC7958902

[37] Sharma BR, Karki R, Kanneganti TD. Role of AIM2 inflammasome in inflammatory diseases, cancer and infection. Eur J Immunol. 2019;49(11):1998–2011. 31372985 10.1002/eji.201848070 PMC7015662

[38] Li N, He F, Gao H, et al. Elevated cell-free fetal DNA contributes to placental inflammation and antiangiogenesis via AIM2 and IFI16 during pre-eclampsia. J Cell Physiol. 2020;235(12):9577–9588. 32383175 10.1002/jcp.29766

